# Entanglement of superconducting qubits via acceleration radiation

**DOI:** 10.1038/s41598-017-00770-z

**Published:** 2017-04-06

**Authors:** L. García-Álvarez, S. Felicetti, E. Rico, E. Solano, C. Sabín

**Affiliations:** 1 Department of Physical Chemistry, University of the Basque Country UPV/EHU, Apartado 644, E-48080 Bilbao, Spain; 2 Laboratoire Matériaux et Phénoménes Quantiques, Sorbonne Paris Cité, Université Paris Diderot, CNRS UMR 7162, 75013 Paris, France; 3 IKERBASQUE, Basque Foundation for Science, Maria Diaz de Haro 3, 48013 Bilbao, Spain; 4 Instituto de Física Fundamental, CSIC, Serrano 113-bis, 28006 Madrid, Spain

## Abstract

We show that simulated relativistic motion can generate entanglement between artificial atoms and protect them from spontaneous emission. We consider a pair of superconducting qubits coupled to a resonator mode, where the modulation of the coupling strength can mimic the harmonic motion of the qubits at relativistic speeds, generating acceleration radiation. We find the optimal feasible conditions for generating a stationary entangled state between the qubits when they are initially prepared in their ground state. Furthermore, we analyse the effects of motion on the probability of spontaneous emission in the standard scenarios of single-atom and two-atom superradiance, where one or two excitations are initially present. Finally, we show that relativistic motion induces sub-radiance and can generate a Zeno-like effect, preserving the excitations from radiative decay.

## Introduction

Circuit Quantum Electrodynamics (cQED)^1–4^ offers both a promising architecture for quantum technologies, such as quantum computers^5, 6^ and simulators^7–9^, and a natural arena for the study of quantum field theory and relativistic effects, either in a direct or simulated fashion^10–14^. For instance, the dynamical Casimir effect (DCE), produced by the modulation of the boundary conditions of the electromagnetic field at relativistic speeds, has been observed in superconducting devices^15–17^. Along these lines, it has been shown that DCE radiation possesses several forms of quantum correlations^18–22^ that can be transferred to superconducting qubits^23, 24^. A related phenomenon is the Unruh effect, where an accelerated detector in vacuum should detect thermal radiation^25, 26^. Recently, some of us have shown how to mimic the generation of acceleration radiation by means of the modulation of the coupling strength of a superconducting qubit^27^, a phenomenon resembling the cavity-enhanced Unruh effect^28–30^. The simulation in a superconducting architecture of both phenomena, DCE and acceleration radiation, relies on the possibility of performing an ultrafast variation of the magnetic flux threading a superconducting quantum interferometric device (SQUID)^31–33^.

**Figure 1:**
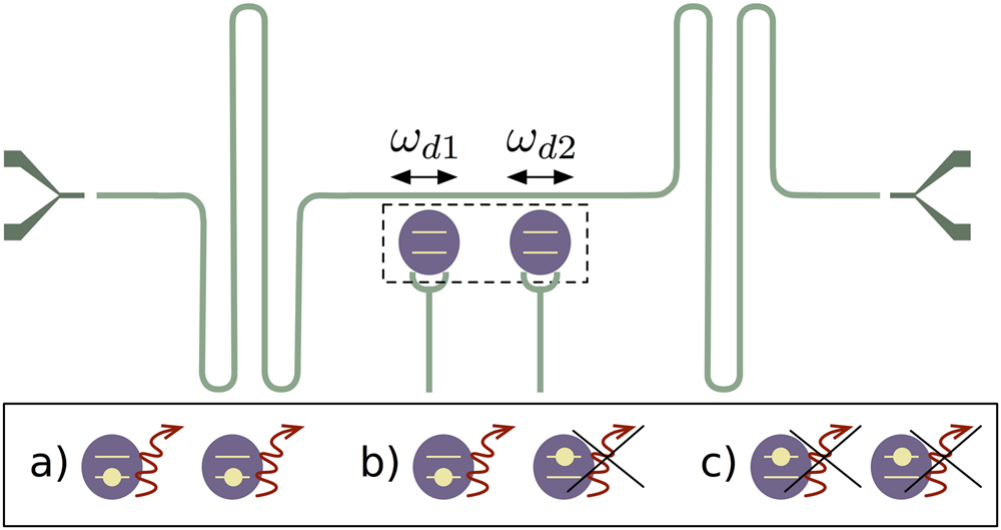
Two superconducting qubits strongly coupled to a single resonator mode and driven with frequencies *ω*_*d*1_ and *ω*_*d*2_ simulating harmonic relativistic speeds. The resonator of length *L*_*c*_ is initially in the vacuum while the qubits, which are located in the middle of the cavity *x* = 0, are initially (**a**) both in the ground state, (**b**) one in the excited state and the other in the ground state, (**c**) both in the excited state. The red wavy arrows indicate emission or absence of emission of photons from the qubits, showing a subradiant behaviour.

In this paper, we consider a superconducting circuit setup in which two superconducting qubits interact with the same resonator mode and effectively move at relativistic speeds, see Fig. 1. The simulation of the relativistic motion of the qubits comes from the modulation of the coupling strength between the qubits and the resonator, which can be interpreted as the qubits movement and activates the counterrotating terms of the quantum Rabi Hamiltonian. We analyse the role of the generated acceleration radiation in several collective properties of the qubits. First, we consider an initial state with no excitations and find the conditions for an efficient generation of stationary entangled states. We find several optimal scenarios for entanglement production: either both qubits move resonantly with the (or half the) natural frequency of the cavity, or one qubit remains static while the other moves at twice the cavity frequency. Second, we analyse the effect of relativistic motion on the spontaneous emission rate when one or two qubit excitations are initially present. Namely, we add the ingredient of relativistic motion to the celebrated Dicke scenario of single-atom and two-atom superradiance^34^, which has been recently implemented in a circuit QED architecture^35^. We will show that the counterrotating dynamics generated by the motion of the qubits tends to suppress the superradiance. Moreover, we find experimental conditions under which the qubit decay is completely frozen, giving rise to a Zeno-like effect induced by the continuous modulation of the coupling strength^36–38^. In this second case, the optimal scenario for the appearance of Zeno-like effect correspond to a synchronised motion of the qubits at twice the frequency of the cavity, which generates an anti-Jaynes-Cummings dynamics in both qubits that prevents them from spontaneous emission.

## Results

### Entanglement and acceleration radiation

The Hamiltonian of the system describes two superconducting qubits of frequency gaps ${\omega }_{\ell }^{q}$ coupled to a single resonator mode of frequency *ω*,

1 $${\mathscr{H}}=\hslash \omega {a}^{ {\dagger } }a+\sum _{\ell =1}^{2}[\frac{\hslash {\omega }_{\ell }^{q}}{2}{\sigma }_{\ell }^{z}+{{\mathscr{H}}}_{I}({x}_{q\ell })].$$

Here, ${\sigma }_{\ell }^{z}$ are Pauli matrices for the qubits, and *a* (*a* ^†^) is an annihilation (creation) operator for the resonator mode. The interaction Hamiltonian depends on the qubits position as

2 $${ {\mathcal H} }_{I}({x}_{q\ell })={g}_{\ell }\,\cos (k{x}_{q\ell }){\sigma }_{\ell }^{x}({a}^{ {\dagger } }+a),$$

with ${g}_{\ell }$ the coupling strength and ${x}_{q\ell }$ the qubit position^39^. In order to simulate the motion of the qubits, which are located in fixed positions, we modulate the coupling with external drivings, such that the interaction Hamiltonian for a qubit reads ${ {\mathcal H} }_{I}({x}_{q\ell })={g}_{\ell }\,\cos ({f}_{0}+{\rm{\Delta }}f\,\cos ({\omega }_{d\ell }t)){\sigma }_{\ell }^{x}({a}^{ {\dagger } }+a)$, and $k{x}_{q\ell }={f}_{0}+{\rm{\Delta }}f\,\cos ({\omega }_{d\ell }t)$. The velocity of the qubits vary harmonically in time, with the maximum value of $\approx \lambda {\omega }_{d}\ell$. For *λ* = 2*L*_*c*_ = 1 cm and ${\omega }_{d}\ell$ = 10 GHz, we reach values of ≈10^8^ m/s = *c*/3.

Initially, we consider the system in the ground state for the qubits and the resonator mode $|{g}_{1}{g}_{2}0\rangle$. In order to determine the degree of entanglement between the qubits after a certain interaction time *T*, we compute the concurrence, which up to second order in perturbation theory with respect to ${g}_{\ell }/\omega$ reads *C* = 2*Max*{|*X*| − *P*_*e*_, 0}^40^. Here, *X* is the amplitude for photon exchange between the qubits, $X=\langle 0|{\mathscr{T}}({{\mathscr{S}}}_{1}^{+}{{\mathscr{S}}}_{2}^{+})|0\rangle$, with ${\mathscr{T}}$ the time-ordering operator. *P*_*e*_ is the probability of emitting a photon, ${P}_{e}=\langle 0|{{\mathscr{S}}}_{1}^{-}{{\mathscr{S}}}_{1}^{+}|0\rangle$, with ${{\mathscr{S}}}_{\ell }^{+}=-\frac{i\,{g}_{\ell }}{\hslash }{\int }_{0}^{T}{e}^{i{\omega }_{\ell }^{q}{t}^{^{\prime} }}dt^{\prime} \cos (k{x}_{q\ell })({e}^{i\omega {t}^{^{\prime} }}{a}^{ {\dagger } }+{e}^{-i\omega {t}^{^{\prime} }}a)dt^{\prime} =-{({{\mathscr{S}}}_{\ell }^{-})}^{ {\dagger } }$. In this configuration, if both qubits are at fixed positions, the emission and photon exchange are counterrotating processes, that is, related to the breakdown of the rotating-wave approximation (RWA). These processes will be significant only for ultrastrong couplings or short interaction times. However, the motion of the qubits can excite these counterrotating terms of the Hamiltonian, giving rise to a sizeable emission of photons by a sort of cavity-enhanced Unruh effect^28^.

We will analyse now under which conditions this phenomenon can be exploited to efficiently generate entanglement between the qubits. We can gain first insights by using analytical techniques. For the sake of simplicity, we assume that ${\omega }_{1}^{q}={\omega }_{2}^{q}={\omega }^{q}$ and *g*_1_ = *g*_2_ = *g*. Moreover, for the sake of simplicity, we consider that the harmonic oscillations of the qubits with frequency *ω*_*d*_ preserve its relative distance, *x*_2_(*t*) − *x*_1_(*t*) = *D* = *λ*/4, where ${x}_{1}(t)=-\frac{D}{2}\mathrm{(1}-\,\cos \,{\omega }_{d}\,t)$, and *λ* is the wavelength associated with the cavity frequency *ω*. Then, we have

3 $$\begin{array}{ccc}X & \simeq  & {g}^{2}{\displaystyle \int }_{0}^{T}d{t}_{2}{\displaystyle \int }_{0}^{{t}_{2}}d{t}_{1}{e}^{i{\rm{\Delta }}{t}_{2}}{e}^{i(2{\omega }^{q}-{\rm{\Delta }}){t}_{1}}\cdot [{J}_{0}({\textstyle \tfrac{\pi }{2}})-2{J}_{2}({\textstyle \tfrac{\pi }{2}})\cos 2{\omega }_{d}t]\\ {P}_{e} & \simeq  & \frac{{g}^{2}}{2}{|{\displaystyle \int }_{0}^{T}dt{e}^{i(2{\omega }^{q}-{\rm{\Delta }})t}[{J}_{0}({\textstyle \tfrac{\pi }{4}})-2{J}_{2}({\textstyle \tfrac{\pi }{4}})\cos 2{\omega }_{d}t+2{J}_{1}({\textstyle \tfrac{\pi }{4}})\cos {\omega }_{d}t]|}^{2},\end{array}$$

where Δ is the detuning between the qubits and the cavity, Δ = *ω* ^*q*^ − *ω*, and *J*_*n*_(*x*) are Bessel functions of the first kind. By inspection of Eq. (3), we observe that both *X* and *P*_*e*_ are, in general, oscillating functions. However, under certain resonant conditions, the oscillations are suppressed and these magnitudes grow monotonically in time. For instance, for negligible detuning Δ = 0, and frequencies *ω*_*d*_ = *ω* = *ω* ^*q*^, we find that $|X|\simeq \frac{{g}^{2}}{2}{J}_{2}(\frac{\pi }{2}){T}^{2}$ and ${P}_{e}\simeq \frac{{g}^{2}}{2}{J}_{2}{(\frac{\pi }{4})}^{2}{T}^{2}$, by keeping only the non-oscillating terms. Since $\frac{{J}_{2}(\frac{\pi }{2})}{{J}_{2}{(\frac{\pi }{4})}^{2}}\gg 1$, the entanglement grows quadratically in time as

4 $$C\simeq {g}^{2}{T}^{2}[{J}_{2}({\textstyle \tfrac{\pi }{2}})-{J}_{2}{({\textstyle \tfrac{\pi }{4}})}^{2}].$$

**Figure 2:**
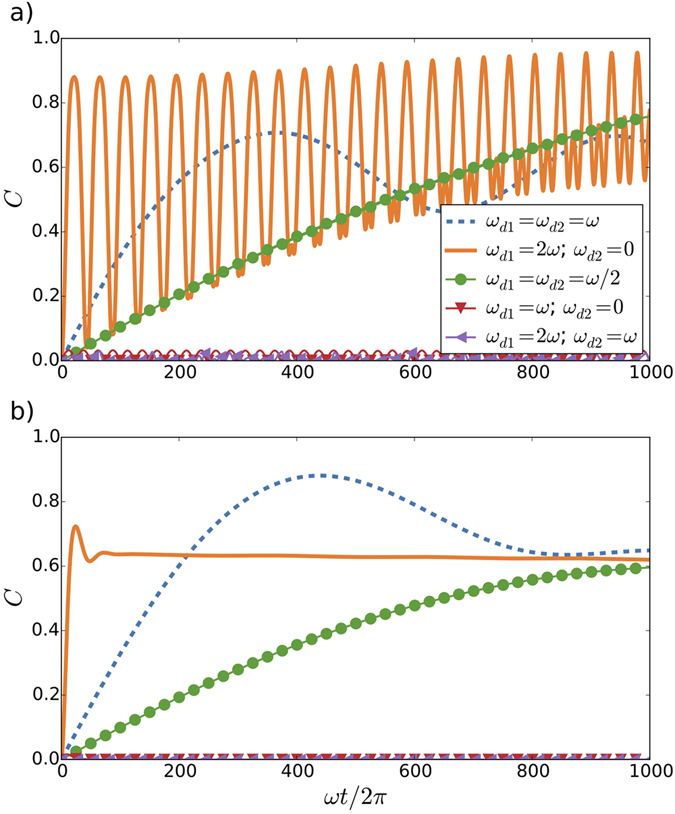
Concurrence *C* of two qubits initially located in the centre of the resonator and oscillating from mirror to mirror with frequencies *ω*_*d*1_ and *ω*_*d*2_, respectively. For coupling constants *g*_1_ = *g*_2_ = *g* = 0.02, initial state $|{g}_{1}{g}_{2}0\rangle$, qubit decay parameter Γ = 0.002, and *T*_2_/*T*_1_ = 0.67, we consider two cavity decay rates: (**a**) *κ* = 0.002, and (**b**) *κ* = 0.2 (bad-cavity limit), in units of *ω*. These numerical results correspond to a broader parameter range, not limited by the perturbative approximation *gT* < 1, which for this case of *g* = 0.02 breaks for ≈8*ωt*/2*π*.

Therefore, we predict an entanglement resonance around *ω*_*d*_ = *ω*. These analytical results are limited by the perturbative approximation employed, which assumes that *gT* < 1. Even in the weak coupling regime, the perturbative approximation would eventually break down. In Fig. 2, we plot the results of numerical simulations which generalise our analytical insights. The dynamics is governed by a master equation where we introduce a cavity decay rate *κ*, a decay parameter Γ accounting for dissipative processes, as well as a decay Γ_*φ*_ for the dephasing of the qubits. The energy relaxation time and phase coherence time are denoted with *T*_1_ = 1/Γ and *T*_2_ = 1/Γ_*ϕ*_, respectively. We consider realistic parameters, achievable with present technology^41^. This allows us to analyse the long-term dynamics of the system and to consider more general types of motion with *ω*_*d*1_ ≠ *ω*_*d*2_, in which the relative distance of the qubits is no longer preserved. Numerical simulations confirm the generation of a high degree of entanglement in the case of *ω*_*d*1_ = *ω*_*d*2_ = *ω*_*d*_ = *ω*, as expected for short-time dynamics. In the long-term dynamics, we observe non-trivial entanglement oscillations, where maximum values are achieved at particular times shown in Fig. 2. Another optimal scenario for entanglement generation appears when one qubit is effectively moving with frequency *ω*_*d*1_ = 2*ω*, and the other remains static, *ω*_*d*2_ = 0. Under these circumstances, the first qubit is ruled by an anti-Jaynes Cummings (anti-JC) dynamics which maximises the counterrotating emission of photons^27^. In this case, the concurrence reaches its maximal value, and the amplitude of initially perfect collapse-revival cycles eventually diminish. Asymptotically, entanglement exhibits small fluctuations around a mean value close to one. Moreover, if we also consider the bad-cavity limit, where $\kappa \gg g\gg {\rm{\Gamma }}$, entanglement oscillations are smoothed out, and highly entangled stationary states are reached, see Fig. 2b. We extend our analysis of the generation of entanglement between both qubits for the case in which the cavity is out of resonance from both drivings and qubits frequencies. Although we have analytically seen that the concurrence increases when the resonant condition is fulfilled, we expect that considering a detuned cavity will enhance the quantum correlation between the qubits (see Supplementary Information).

### Single-atom superradiance and Zeno-like effect

**Figure 3:**
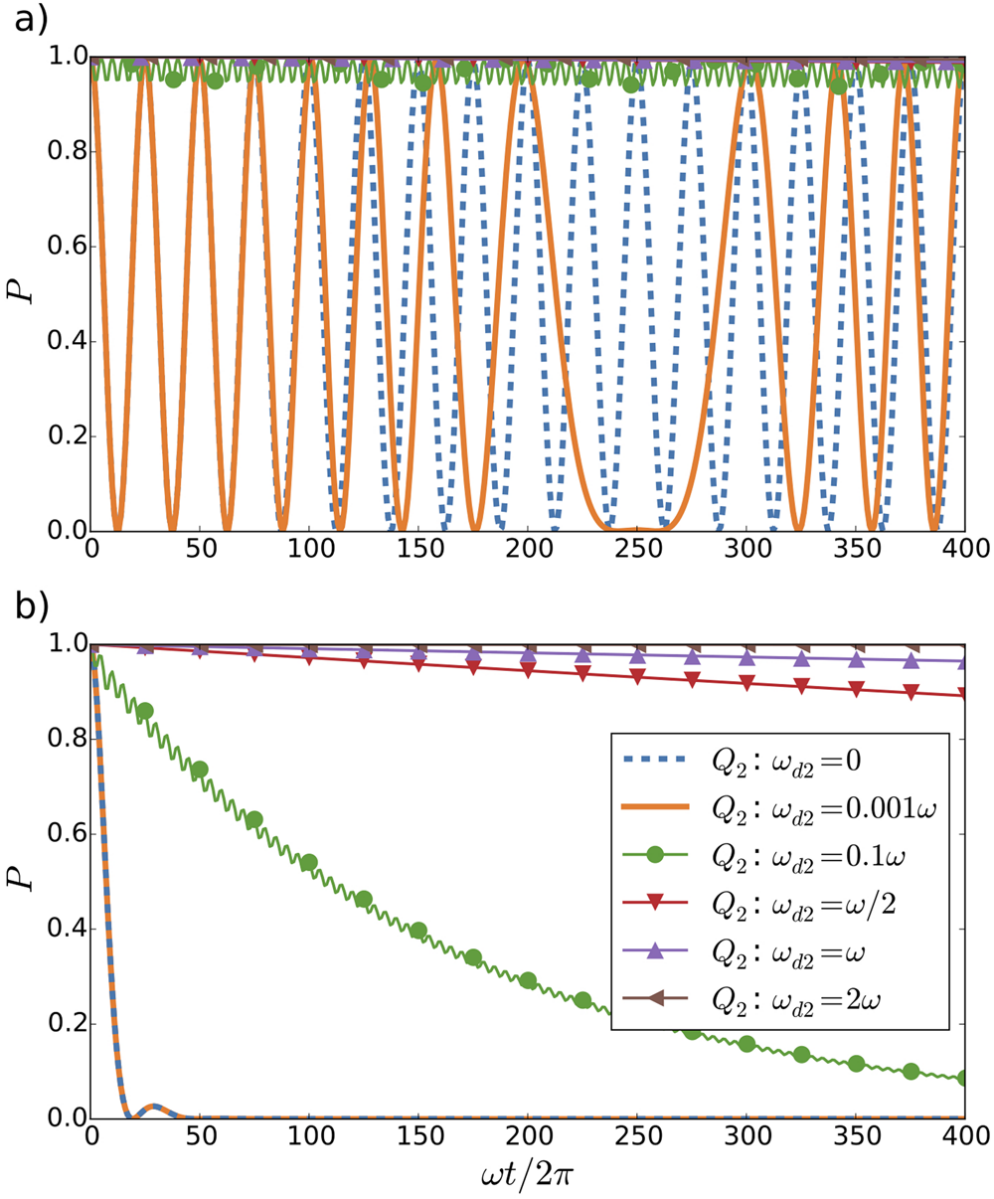
Motion effects in single-atom superradiance, observed in the probability *P* of excitation of the second qubit *Q*_2_. We consider the first qubit *Q*_1_ decoupled, *g*_1_ = 0, and the second moving with frequency *ω*_*d*2_, for an initial state $|{g}_{1}{e}_{2}0\rangle$. We show the behaviour for different velocities of the second qubit, ranging from the static case, *ω*_*d*2_ = 0, to a velocity of *ω*_*d*2_ = 2*ω*. For a coupling constant *g*_2_ = 0.02, a qubit decay parameter Γ = 0.002 and *T*_2_/*T*_1_ = 0.67, we consider two cavity decay rates: (**a**) *κ* = 0.002, and (**b**) *κ* = 0.2 (bad-cavity limit), in units of *ω*.

In his seminal work, Dicke showed that the decay of atomic emitters is enhanced by the presence of other atomic emitters^34^. The simplest case, called single-atom superradiance, consists of a single emitter in an excited state influenced by the proximity of another emitter, even if the latter is in the ground state. This *gedanken experiment* has been recently realised in a circuit QED architecture in the bad-cavity limit^35^. Here, we analyse the effects of relativistic motion in this scenario. To this end, we consider the initial state $|{g}_{1}{e}_{2}0\rangle$, and discuss, firstly, the effects of the relativistic motion of the second qubit in its decay, and, secondly, the effects of the presence of the first qubit in the decay of the second one. We observe that the relativistic motion of the second qubit, encoded in *ω*_*d*2_, tends to inhibit its decay leading to a decreased emission rate, known as sub-radiance, see Fig. 3. Again, we can get some insight on the system dynamics from first-order analytical computations. Since in this case the qubit is initially excited, the probability of emitting a photon is not given by Eq. (3). In particular, in the resonant case (Δ = 0), we have ${P}_{e}={g}_{2}^{2}{|{\int }_{0}^{T}dt\cos (k{x}_{q2}(t))|}^{2}$. Then, considering the trajectories for both qubits such that ${x}_{q\ell }={L}_{c}/2+{L}_{c}/2\,\cos ({\omega }_{d\ell }\,t)$ we get ${P}_{e}\simeq 4{g}_{2}^{2}{J}_{1}^{2}(\pi /\mathrm{2)}{\sin }^{2}({\omega }_{d2}T)/{\omega }_{d2}^{2}\simeq {g}_{2}^{2}{\sin }^{2}({\omega }_{d2}T)/{\omega }_{d2}^{2}$. This means that for a static qubit -*ω*_*d*2_ close to 0-, the probability of emission grows quadratically in time ${P}_{e}\simeq {g}^{2}{T}^{2}$, while for frequencies of motion significantly different from 0 the probability oscillates with an amplitude which decreases with *ω*_*d*2_. Therefore, for large enough *ω*_*d*2_ the probability of emission is suppressed, as can be seen in Fig. 3. Note that the maximum acceleration of the qubit motion is proportional to ${\omega }_{d2}^{2}$. Thus, the larger the acceleration is, the larger the suppression of the probability of emission. Then, the sub-radiance can be seen as another relativistic effect, hitherto unexplored. At first glance, this subradiant dynamics might look surprising, since relativistic accelerated motion is typically associated to the emission of photons. However, note that both phenomena, Unruh effect and subrradiance, are activated by the counterrotating terms of the Hamiltonian, which become dominant for large enough *ω*_*d*2_ associated with relativistic motion. While non-RWA dynamics gives rise to emission of photons when the qubit and cavity start in the ground state, in the present case the initial state $|{e}_{2}0\rangle$ would be stationary in the presence of only non-RWA terms. The decay dynamics of the second qubit is effectively frozen, that is, we observe a Zeno-like effect generated by the continuous modulation of the qubit-cavity coupling strength, which has an effect similar to a continuous monitoring of the system^36–38^.

**Figure 4:**
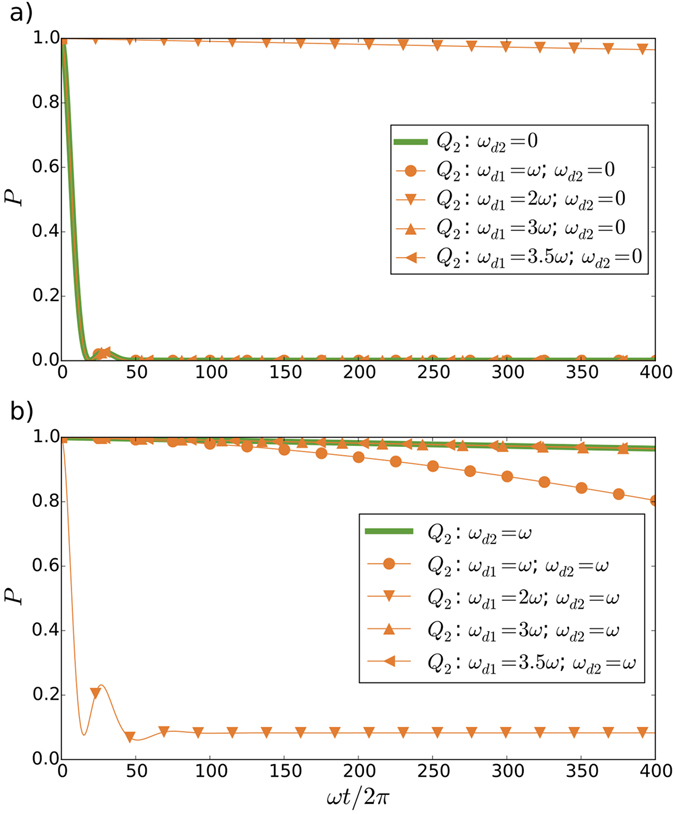
Motion effects in single-atom superradiance, observed in the probability *P* of excitation of the second qubit *Q*_2_. We consider the influence of the movement of the first qubit *Q*_1_ by analysing different velocities *ω*_*d*1_ and the decoupled case, *g*_1_ = 0. The second qubit is moving with frequency *ω*_*d*2_, for an initial state $|{g}_{1}{e}_{2}0\rangle$. We compute for a coupling constant *g*_1_ = *g*_2_ = 0.02 in the cases with the first qubit coupled, a qubit decay parameter Γ = 0.002 and *T*_2_/*T*_1_ = 0.67, and a cavity decay rate *κ* = 0.2 (bad-cavity limit), in units of *ω*. We show the modification in the behaviour in the case of the second qubit (**a**) static *ω*_*d*2_ = 0, and (**b**) moving with *ω*_*d*2_ = *ω*.

Not only the relativistic motion of the second qubit, encoded in *ω*_*d*2_, tends to inhibit its decay, but also the motion of the first qubit, *ω*_*d*1_, has a significant effect on the emission rate for certain values of *ω*_*d*1_. In order to analyse this influence, we compare in Fig. 4 the probability of excitation of the second qubit for the case in which the first qubit is decoupled, and the case in which it is coupled moving at different relativistic speeds, *ω*_*d*1_. Firstly, we consider the extreme case with the second qubit static, *ω*_*d*2_ = 0, and we observe that a first qubit relativistic speed *ω*_*d*1_ = 2*ω* leads to a decreased emission rate, known as sub-radiance, whereas for other combinations of frequencies, the behaviour of the probability of excitation of the second qubit remains unaltered. Secondly, we analyse the case with the second qubit moving with *ω*_*d*2_ = *ω*, and observe a dramatic change in the decay rate of the second qubit for the same frequency *ω*_*d*1_ = 2*ω*, as in the previous case. We notice that the emission rate of the second qubit is also slightly modified for a relation of frequencies *ω*_*d*1_ = *ω*_*d*2_ = *ω* that generates entanglement. However, a further analysis in the relation of the generated entanglement and single-atom superradiance allows us to discard drastic influences of the former in the decay rate (see Supplementary Information). We also interpret the frozen dynamics of the second qubit for *ω*_*d*2_ = *ω*, and for *ω*_*d*2_ = *ω* and *ω*_*d*1_ = 2*ω*, as a Zeno-like effect^36–38^.

**Figure 5:**
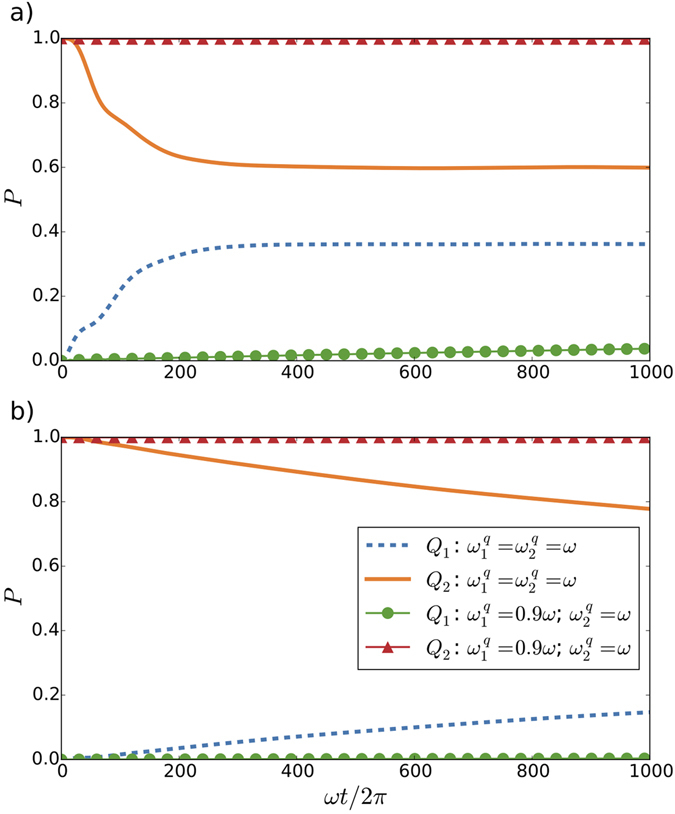
Zeno-like effect in the probability *P* of excitation of two qubits moving with the same frequency *ω*_*d*1_ = *ω*_*d*2_ = 2*ω* for different amplitudes of oscillation for the first qubit *Q*_1_. We consider the initial state $|{g}_{1}{e}_{2}0\rangle$, a cavity decay rate *κ* = 0.1, coupling constants *g*_1_ = *g*_2_ = *g* = 0.01, and qubit decay parameter Γ = 0.001 and *T*_2_/*T*_1_ = 0.67, in units of *ω*, and that the first qubit is initially placed at *L*_*c*_/4, with *L*_*c*_ the cavity length. We show the excitation probabilities of both qubits, *Q*_1_ and *Q*_2_, with frequencies ${\omega }_{1}^{q}={\omega }_{2}^{q}=\omega$, and adding a detuning Δ = 0.1*ω* between the first qubit and the cavity, for an amplitude of motion of the first qubit (**a**) *L*_c_/4, and (**b**) *L*_*c*_/16.

We extend our analysis to other scenarios by considering different frequencies and initial conditions. We confirm the Zeno-like effect when we reduce the oscillation amplitude and the excitation probability of the first qubit, which enhances even more the effect on the second qubit (see Fig. 5). Two-atom superradiance is a collective effect consisting in an enhancement of the decay rate of two emitters with respect to their individual ones^34^. We consider the effects of relativistic motion on the decay of two artificial atoms, $|{e}_{1}{e}_{2}0\rangle$ and observe a sub-radiance phenomenon, as in the case of single-atom superradiance (see Supplementary Information).

### Implementation in superconducting circuits

The model described in Eq. (1) can be implemented in a circuit QED architecture^42^, using a single-mode transmission line resonator (TLR) interacting with two tunable-coupling superconducting qubits. In order to observe all the phenomenology so far described in a single device, it is required independent tuning of the qubit transition frequencies and of the qubit-cavity coupling strengths. Tunable coupling superconducting qubits^41, 43, 44^, coupled to a TLR and tuning of effective couplings over nanosecond time-scale^45, 46^ have been proven in circuit QED architectures. We provide a more detailed discussion of the implementation with actual values for the parameters in the Supplementary Information.

## Discussion

We have proposed a possible realisation in which simulated relativistic motion generates true entanglement between artificial atoms, while protecting them from spontaneous emission in a Zeno-like effect. A natural extension would be to consider the effects of multi-atom relativistic motion, with a study of superradiant phase transition. Both the ability of generating entanglement and state protection may pave the way for new applications in superconducting quantum technologies.

## Electronic supplementary material

Supplementary Information for Entanglement of superconducting qubits via acceleration radiation
