# Supplementary material for: Entanglement of superconducting qubits via acceleration radiation
Source: Sci Rep. 2017 Apr 6;7:657. doi: 10.1038/s41598-017-00770-z (PMC5429651; doi:10.1038/s41598-017-00770-z)
Supplement: Supplementary file 1 — Supplementary Information for Entanglement of superconducting qubits via acceleration radiation [file 41598_2017_770_MOESM1_ESM.pdf]

Supplementary Information for
Entanglement of superconducting qubits via acceleration radiation

Laura García-Álvarez,[1] Simone Felicetti,[2] Enrique Rico,[1,3] Enrique Solano,[1,3] and Carlos Sabín[4]

[1]Department of Physical Chemistry, University of the Basque Country UPV/EHU, Apartado 644, E-48080 Bilbao, Spain
[2]Laboratoire Matériaux et Phénomènes Quantiques, Sorbonne Paris Cité, Université Paris Diderot, CNRS UMR 7162, 75013, Paris, France
[3]IKERBASQUE, Basque Foundation for Science, Maria Diaz de Haro 3, 48013 Bilbao, Spain
[4]Instituto de Física Fundamental, CSIC, Serrano 113-bis 28006 Madrid, Spain

S1. HIGHER CONCURRENCE WITH OFF-RESONANT CAVITY

In this section, we extend the analysis of concurrence generation between the pair of moving qubits in the cavity, for the case in which the cavity is detuned from the qubits. We observe that for some specific times in the evolution of the system, the correlation between the qubits is enhanced reaching values close to one. This fact can be explained by considering that the cavity just virtually mediates the interaction between the qubits. As it can be seen in Fig. S1, this expected feature appears for certain instants of the system time evolution, in a good cavity limit, where the concurrence shows an oscillatory behavior.

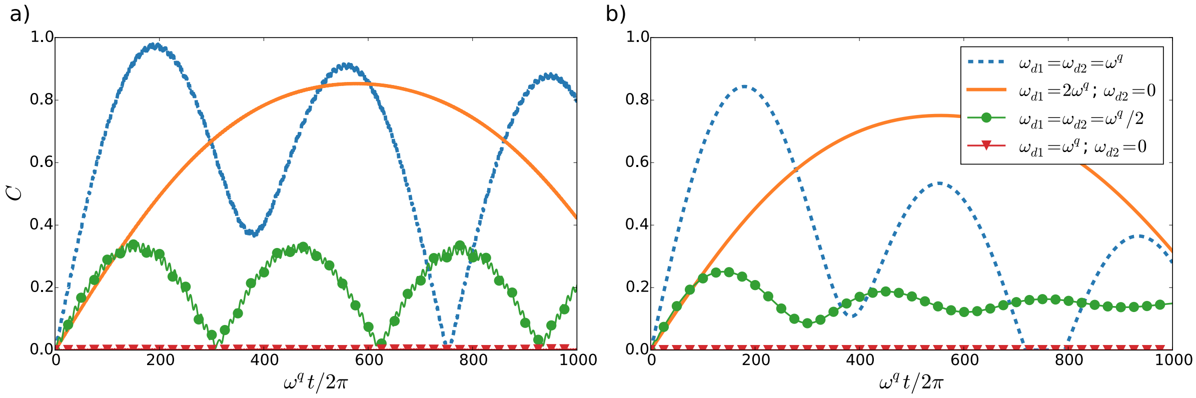

FIG. S1: Concurrence C of two qubits initially located in the center of the resonator and oscillating with frequencies ω_{d1} and ω_{d2}, respectively. We consider coupling constants $g_1 = g_2 = g = 0.02$, a qubit decay parameter $\Gamma = 0.002$ and $T_2/T_1 = 0.67$, and an off-resonant cavity with $\omega = 0.4$, in units of ω^q. For initial state $|g_1\, g_2\, 0\rangle$, we compute for two regimes of decoherence, characterized by a cavity decay rate a) $\kappa = 0.002$, and b) $\kappa = 0.2$ (bad-cavity limit), again in units of ω^q.

S2. RELATION OF CONCURRENCE AND SINGLE-ATOM SUPERRADIANCE

We have explored numerically the influence of the movement of the first qubit in the emission rate of the second qubit in Fig. 4 of the main text. In order to better interpret the possible relation between the generated entanglement and the influence of the first qubit, we show in Fig. S2 the concurrence for the relation of frequencies ω_{d1} and ω_{d2} analyzed in Fig. 4. We conclude that a combination of frequencies generating entanglement is not relevant for observing significant changes in the probability of the decay of the second qubit, although for $\omega_{d1} = \omega_{d2} = \omega$ it results in a slight modification of the decay rate.

S3. TWO-ATOM SUPERRADIANCE AND COLLECTIVE ZENO-LIKE EFFECT

We consider the phenomenon of two-atom superradiance in our setup. We compare in Fig. S3 the decay rate of both qubits moving with relativistic velocities, encoded in ω_{d1} and ω_{d2}, with respect to their individual decay rates, i.e., with respect to the decay of a single static qubit. We observe that the relativistic motion alters the emission rate and reduces drastically for both qubits the probability of decay, leading to a sub-radiance phenomenon. Both in the good cavity and bad cavity limit, we observe a collective Zeno-like effect, namely the effective freezing of the decay of both qubits. This ties in with the prediction of the

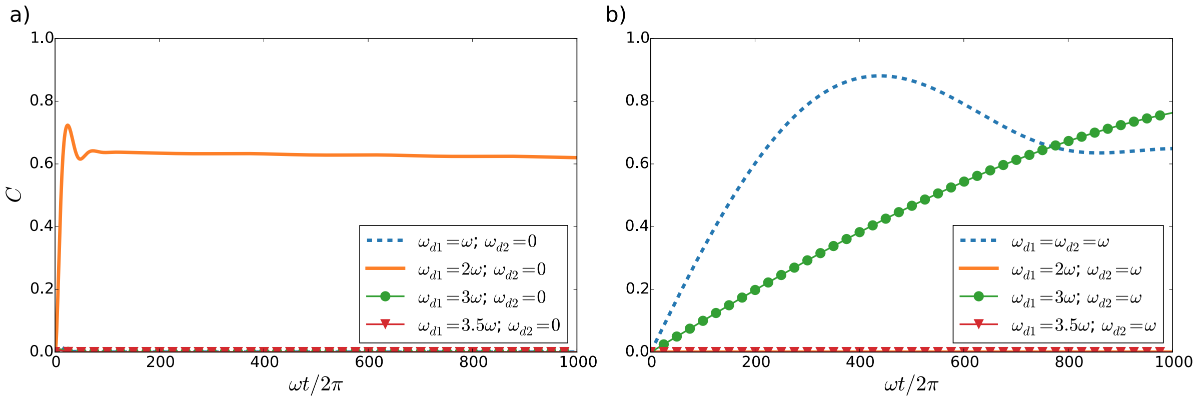

FIG. S2: Concurrence C of two qubits moving with different velocities ω_{d1} and ω_{d2}, previously analyzed in the light of single-atom superradiance. We consider a coupling constant $g_1 = g_2 = 0.02$, a qubit decay parameter $\Gamma = 0.002$ and $T_2/T_1 = 0.67$, and a cavity decay rate $\kappa = 0.2$ (bad-cavity limit), in units of ω. We show results for different velocities ω_{d1} of the first qubit, when the second qubit is a) static $\omega_{d2} = 0$, and b) moving with $\omega_{d2} = \omega$.

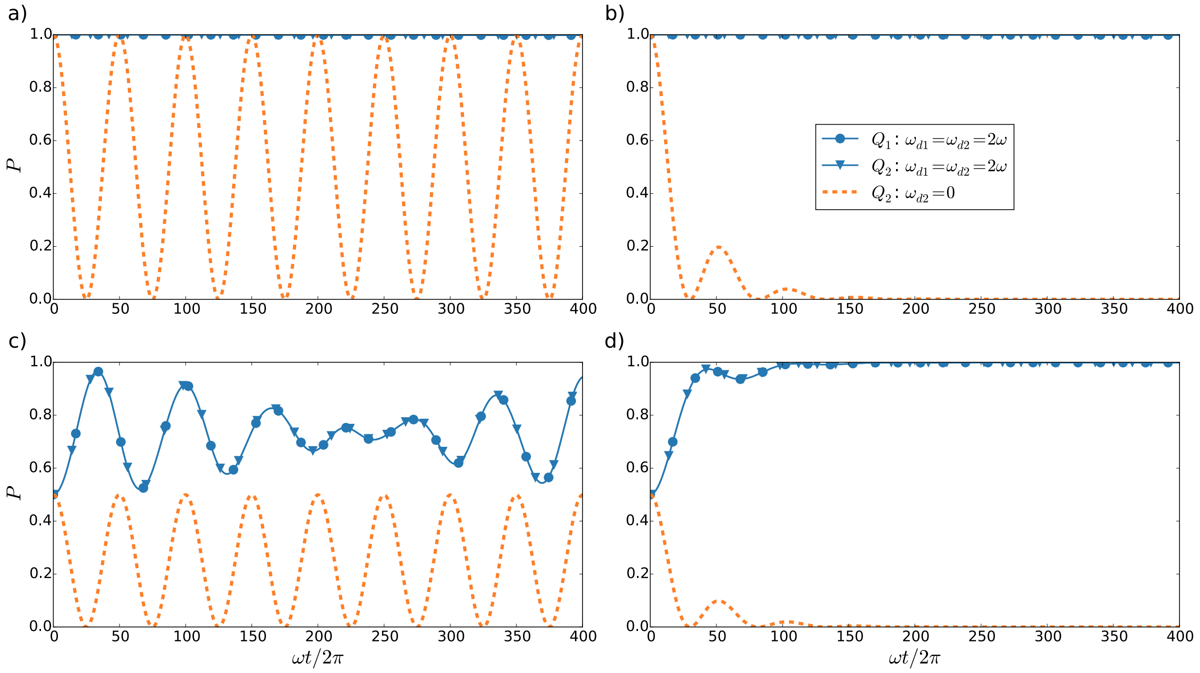

FIG. S3: Probability P of excitation of two qubits, Q_1 and Q_2, moving with different frequencies ω_{d1}, ω_{d2}, and a qubit decay parameter $\Gamma = 0.001$ and $T_2/T_1 = 0.67$, in units of ω. a), b) Both qubits are initially excited. c), d) The initial state of the qubits is $|+_1\ +_2\rangle$. a) , c) $\kappa = 0.001$, and b), d) $\kappa = 0.1$ (bad-cavity limit), in units of ω. All figures show the case of both qubits coupled to the cavity with coupling strengths $g_1 = g_2 = g = 0.01$, $\omega_{d1} = \omega_{d2} = 2\omega$, and the case of an uncoupled first qubit $g_1 = 0$, with a static second qubit $\omega_{d2} = 0$ with $g_2 = 0.01$.

expected anti-JC dynamics at this particular driving frequency [1]. Finally, if we consider a different initial state where both qubits are in a superposition of their ground and excited states, $|+_1\ +_2\,0\rangle$, with $|+\rangle = 1/\sqrt{2}(|e\rangle + |g\rangle)$, we observe that the qubits are driven fast to their excited states, and the ensuing dynamics follows then the one already observed for an initial $|e_1\, e_2\rangle$.

S4. IMPLEMENTATION DETAILS

With the aim of providing a specific implementation proposal, we focus on a three island superconducting qubit [2, 3]. The qubit scheme consists in two shunted SQUID loops described by a model composed of two interacting anharmonic oscillators, whose dynamics can be effectively restricted to their two lowest eigenstates. Independent control over the corresponding transition frequencies results in a completely tunable qubit. We denote $|0\rangle_a$ the ground and $|1\rangle_a$ the first excited state of the oscillator a, and we call ω_a the corresponding transition frequency. Same notation will apply for the oscillator b. The qubit logical levels are given by the ground $|E_0\rangle$ and first-excited $|E_1\rangle$ states of the collective system, whose structure depends on the ratio between the frequencies ω_i. When the two anharmonic oscillators are detuned, the lowest collective eigenstates are given by $|E_0\rangle = |0\rangle_a|0\rangle_b$ and $|E_1\rangle = |1\rangle_a|0\rangle_b$, where we set $\omega_a < \omega_b$. In this configuration, the qubit is strongly coupled to the TLR. On the other hand, when the two anharmonic oscillators are nearly degenerate $\omega_a \approx \omega_b$, the first collective excited state is given by $|E_1\rangle = (|1\rangle_a|0\rangle_b - |0\rangle_a|1\rangle_b)/\sqrt{2}$. Such state corresponds to an antiparallel configuration of the dipoles of the SQUID loops, hence creating a quadrupolar moment that does not couple with the resonator. Swapping between the two collective energy-configurations, the effective coupling can be continuously tuned in real time, without exciting higher energy levels [4], which are detuned by at least 1 GHz from the primary qubit transition [2]. Using this scheme, tuning of the qubit-cavity coupling strength has been experimentally achieved in the range from 40 MHz to less than 200 KHz [3].

System initialization is trivial in all the cases considered in this manuscript, as only ground-state cooling and single-qubit gates are required. Fast read-out of the qubit state can be implemented [5] driving a transition to the second-excited collective state $|E_1\rangle \to |E_2\rangle$. In the resonant case, $\omega_a = \omega_b$, such state is given by $|E_2\rangle = (|1\rangle_a|0\rangle_b + |0\rangle_a|1\rangle_b)/\sqrt{2}$ and it is strongly coupled to the resonator. The qubit excitation states can be then individually obtained measuring the state-dependent shift on the TLR resonant frequency.

[1] Felicetti, S. *et al.* Relativistic motion with superconducting qubits. *Phys. Rev. B* **92,** 064501 (2015).

[2] Gambetta, J. M., Houck, A. A. & Blais, A. Superconducting qubit with Purcell protection and tunable coupling. *Phys. Rev. Lett.* **106,** 030502 (2011).

[3] Srinivasan, S. J., Hoffman, A. J., Gambetta, J. M. & Houck, A. A. Tunable coupling in circuit quantum electrodynamics using a superconducting charge qubit with a V-shaped energy level diagram. *Phys. Rev. Lett.* **106,** 083601 (2011).

[4] Mezzacapo, A., Lamata, L., Filipp, S. & Solano, E. Many-body interactions with tunable-coupling transmon qubits. *Phys. Rev. Lett.* **113,** 050501 (2014).

[5] Zhang, G., Liu, Y., Raftery, J. J. & Houck, A. A. Suppression of photon shot noise dephasing in a tunable coupling superconducting qubit. *npj Quantum Information* **3,** 1 (2017).
